# Permanent pacemaker rate following Commando and Hemi-Commando procedures: a systematic review and meta-analysis

**DOI:** 10.3389/fcvm.2026.1854238

**Published:** 2026-06-23

**Authors:** Yang Zhang, Ren Zhu, Guanshui Yu, Lian Hu, Yong Cao

**Affiliations:** Department of Cardiovascular Surgery, The People’s Hospital of Gaozhou, Maoming, China

**Keywords:** commando procedure, heart block, meta-analysis, permanent pacemaker, systematic review

## Abstract

**Objective:**

The Commando procedure (double-valve replacement with intervalvular fibrous body reconstruction) is associated with a high risk of postoperative atrioventricular block requiring permanent pacemaker implantation. However, precise estimates vary widely. This systematic review and meta-analysis aimed to determine the pooled permanent pacemaker rate after Commando and Hemi-Commando procedures.

**Methods:**

PubMed, Embase, Web of Science, and Cochrane CENTRAL were searched from inception to April 2, 2026. Studies reporting permanent pacemaker rates after Commando or Hemi-Commando surgery (sample size ≥ 5) were included. A random-effects model was used to pool proportions, with logit transformation. Subgroup analyses were performed by procedure type. Sensitivity analyses and publication bias assessment (Egger's test) were conducted.

**Results:**

Twelve studies with 13 data points (797 patients) were included. The pooled permanent pacemaker rate was 22.3% (95% confidence interval: 16.1%–30.0%), with substantial heterogeneity (*I²* = 73.7%). Subgroup analysis showed that the Chimney Commando modification had a significantly lower rate (6.3%, 95% CI: 3.2%–12.1%) compared with the traditional Commando procedure (28.3%, 95% CI: 21.2%–36.7%; *p* for subgroup difference < 0.001). Sensitivity analyses confirmed the robustness of the findings. Egger's test indicated potential small-study effects (*p* = 0.0041).

**Conclusions:**

One in five patients undergoing Commando surgery requires a permanent pacemaker. Preliminary evidence suggests that the Chimney Commando modification may be associated with a substantially lower risk (6.3% vs. 28.3%). Based on these preliminary findings, the Chimney technique might be a reasonable option in carefully selected patients with small annuli or those undergoing redo surgery, where conduction injury risk is inherently high. However, given the limited evidence, this recommendation should be considered hypothesis-generating. Future prospective comparative studies are needed to validate these hypothesis-generating findings.

**Systematic Review Registration:**

https://osf.io/sgd2e/.

## Introduction

The Commando procedure, also known as intervalvular fibrous body (IFB) reconstruction combined with aortic and mitral valve replacement, is a complex operation indicated for destructive infective endocarditis, extensive mitral annular calcification, radiation-induced heart disease, and redo double-valve surgery (1, 2). Despite its life-saving potential, the procedure carries a substantial risk of postoperative atrioventricular (AV) block requiring permanent pacemaker implantation (3). Several risk factors for pacemaker implantation after mitral valve surgery have been identified, including advanced age, female sex, and preoperative renal dysfunction (4).

A previous systematic review and meta-analysis summarized early and long-term outcomes of Commando surgery, reporting a pooled pacemaker rate of 25.1% (5). Recent narrative reviews have summarized the technical evolution and expanding indications of the Commando procedure (6). However, that analysis included only nine studies and did not specifically evaluate differences between surgical modifications such as the Chimney Commando technique. Given the growing number of recent reports, an updated and focused synthesis is warranted.

Therefore, we conducted this systematic review and meta-analysis to provide a precise, contemporary estimate of permanent pacemaker risk after Commando and Hemi-Commando procedures and to explore potential sources of heterogeneity, including procedure type.

## Methods

This systematic review and meta-analysis was pre-registered on the Open Science Framework (osf.io/sgd2e). Reporting follows the PRISMA 2020 guidelines (7).

### Search strategy

We searched PubMed, Embase, Web of Science, and Cochrane CENTRAL from database inception to April 2, 2026, without language restrictions in the search strategy. However, due to resource constraints, only articles published in English were included after full-text screening. The search combined terms for “Commando procedure”, “intervalvular fibrous body”, “aorto-mitral curtain”, and “double valve replacement”. The complete search strings for each database are provided in Supplementary Table S1. An example PubMed query is shown below:

“(“Commando procedure”[Title/Abstract] OR “Commando operation”[Title/Abstract] OR “UFO procedure”[Title/Abstract] OR “Intervalvular Fibrous Body Reconstruction”[Title/Abstract] OR “Aortic Mitral Curtain Reconstruction”[Title/Abstract] OR “Hemi-Commando”[Title/Abstract] OR “Modified Commando”[Title/Abstract] OR “Root-Commando”[Title/Abstract])”

Reference lists of included articles and relevant reviews were hand-searched.

### Eligibility criteria

#### PICOS framework

This systematic review was conducted following the PICOS framework: Population – patients undergoing Commando or Hemi-Commando surgery; Intervention—Commando or Hemi-Commando procedure (including Chimney Commando modification); Comparator—not applicable for single-arm studies; Outcomes—permanent pacemaker implantation rate; Study design—retrospective cohorts, prospective cohorts, or case series (sample size ≥ 5).

Studies were included if they: (1) reported original data (retrospective cohort, prospective cohort, or case series) on patients undergoing Commando or Hemi-Commando surgery; (2) clearly reported the number of permanent pacemaker implantations and the total number of patients; (3) had a sample size of at least 5 patients; and (4) were published as full-text articles in English. We excluded case reports (*n* < 5), reviews, editorials, technical notes, conference abstracts, and studies with overlapping patient populations (only the largest or most recent from the same institution was retained).

### Study selection and data extraction

Two reviewers independently screened titles/abstracts and full texts. Disagreements were resolved by consensus. Data were extracted using a standardized form: first author, year, procedure type (Commando, Hemi-Commando, Chimney Commando, mixed, pediatric), number of pacemaker events, total patients, and surgical indications.

### Risk of bias assessment

Two reviewers independently assessed the methodological quality of the included case series using the JBI critical appraisal tool for case series studies (8). The tool consists of 10 questions addressing internal validity, confounding, selection bias, information bias, and clarity of reporting. Disagreements were resolved by consensus.

### Statistical analysis

The primary outcome was the pooled permanent pacemaker rate. A random-effects model (restricted maximum-likelihood estimator for *τ*²) was used. The effect size was the logit-transformed proportion, with continuity correction (0.5) for zero-event studies. Heterogeneity was quantified using *I²* and *τ²*, with *I²* > 50% considered substantial. Subgroup analyses were performed by procedure type (Commando, Hemi-Commando, Chimney Commando, mixed, pediatric). Sensitivity analyses included leave-one-out, fixed-effect model, exclusion of small studies (*n* < 20), exclusion of pediatric study, and exclusion of extreme-rate studies (Davierwala 2020 and Yang 2023). Publication bias was assessed using funnel plot and Egger's linear regression test. All analyses were performed using R (version 4.5.2) with the meta package. Because the time window for pacemaker ascertainment varied across studies (in-hospital, 30-day, or entire follow-up), this could contribute to the observed heterogeneity. Meta-regression was considered to explore potential sources of heterogeneity, but it was not performed because the number of studies in the relevant subgroups (e.g., Commando subgroup, *n* = 7) was too small to provide reliable estimates (a minimum of 10 studies is generally recommended).

## Results

### Study selection and characteristics

**Figure 1:**
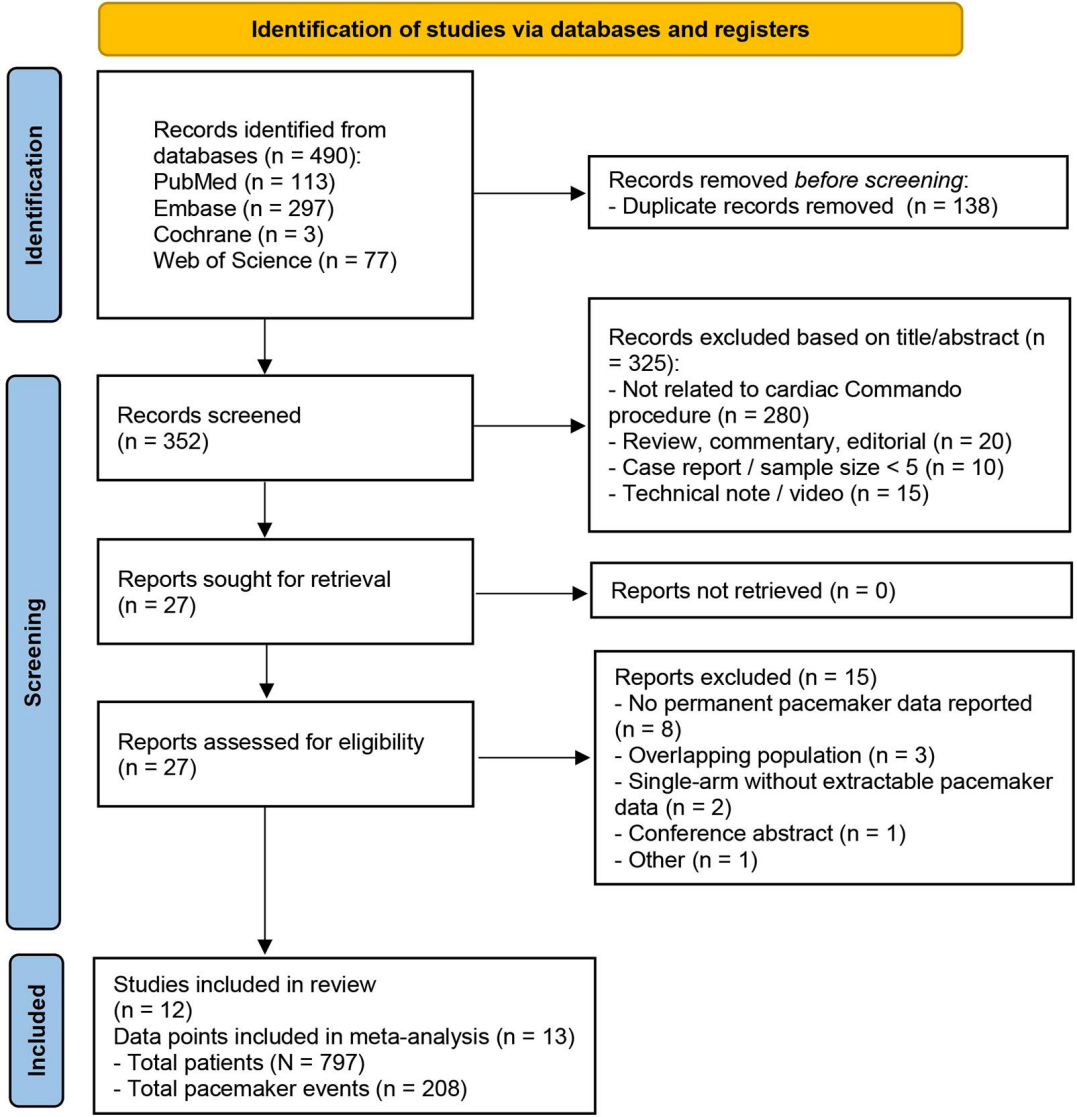
PRISMA 2020 flow diagram showing the study selection process.

The PRISMA flow diagram is shown in Figure 1. A total of 490 records were identified; after duplicate removal and screening, 27 full-text reports were assessed. Fifteen were excluded (no pacemaker data, overlapping populations, conference abstract, etc.), leaving 12 studies with 13 data points (Navia 2019 contributed two data points: Commando and Hemi-Commando) (3, 9–18). Table 1 summarizes the characteristics of the included studies. The 13 data points comprised 797 patients and 208 pacemaker events. Sample sizes ranged from 5 to 182 patients. Indications included infective endocarditis, calcific degeneration, redo surgery, and small annuli.

**Table 1:** Characteristics of included studies.

First author	Year	Procedure type	Pacemaker (*n*)	Total (*N*)	Rate (%)	Indication/notes^§^
Forteza-Gil A	2025	Mixed (non-ROOT + ROOT)	19	78	24.4	IE/Commando variants
Liu H	2025	Chimney Commando	7	101	6.9	Redo DVR, small annuli
Brown JA	2024	Commando	4	21	19.0	Mixed (IE 52.4%)
Bojko M	2024	Commando	8	41	19.5	Mixed (IE 61%)
Vobornik M	2023	Mixed (Commando + Hemi-Commando)	6	20	30.0	IE
Yang M	2023	Chimney Commando	1	30	3.3	Small annuli
Kinami H	2023	Commando (pediatric)	0	5	0.0	Congenital heart disease
David TE	2022	Commando	60	182	33.0	Mixed (calcification, abscess, redo, PPM)
Davierwala PM	2020	Commando	53	127	41.7	IE with IFB destruction
Jiang X	2020	Commando	1	14	7.1	IE
Navia JL	2019	Commando	28	86	32.6	IE/DVR + IFB reconstruction
Navia JL	2019	Hemi-Commando	13	52	25.0	IE/AVR + MV repair + IFB reconstruction
Forteza A	2015	Commando	8	40	20.0	IE (*n* = 26) + calcification (*n* = 14)

IE, infective endocarditis; DVR, double valve replacement; IFB, intervalvular fibrous body; AVR, aortic valve replacement; MV, mitral valve; PPM, patient-prosthesis mismatch.

§ Indication for pacemaker implantation was reported only in three studies: all were complete or high-degree atrioventricular block. The remaining studies did not specify the indication.

### Baseline characteristics of included patients

The baseline characteristics of the 797 patients across the 12 studies are summarized in Supplementary Table S2. The mean age of patients ranged from 52 to 67 years across studies. The proportion of male patients varied between 31.7% and 90.0%. Infective endocarditis was the primary indication for surgery in 61% of patients (range across studies: 13%–100%). A history of prior cardiac surgery was present in 69% of patients (range: 7.1%–82%). EuroSCORE II or logistic EuroSCORE was reported in only five studies, with mean values ranging from 7.4% to 53.0%, reflecting the high-risk nature of the cohort. STS score was not reported in any included study. These data should be considered when interpreting the generalizability of the pooled pacemaker rate.

### Risk of bias assessment

The methodological quality of the 12 included studies was assessed using the JBI critical appraisal tool for case series (10 items). The results are summarized in Supplementary Table S3. Across all studies, the median total score was 9 (range: 8–10), indicating moderate to good methodological quality. Common strengths included clear definition of inclusion criteria, use of standard outcome measures (pacemaker implantation), and appropriate statistical analysis. Common weaknesses were the lack of consecutive patient enrollment (e.g., Bojko 2024, Forteza 2015) and incomplete reporting of follow-up duration (e.g., Brown 2024, Vobornik 2023, Yang 2023, Jiang 2020). The predominantly retrospective design and absence of control groups limit the certainty of the pooled estimates, but the overall quality of the included studies is acceptable for a systematic review of rare surgical procedures.

### Pacemaker rate

**Figure 2:**
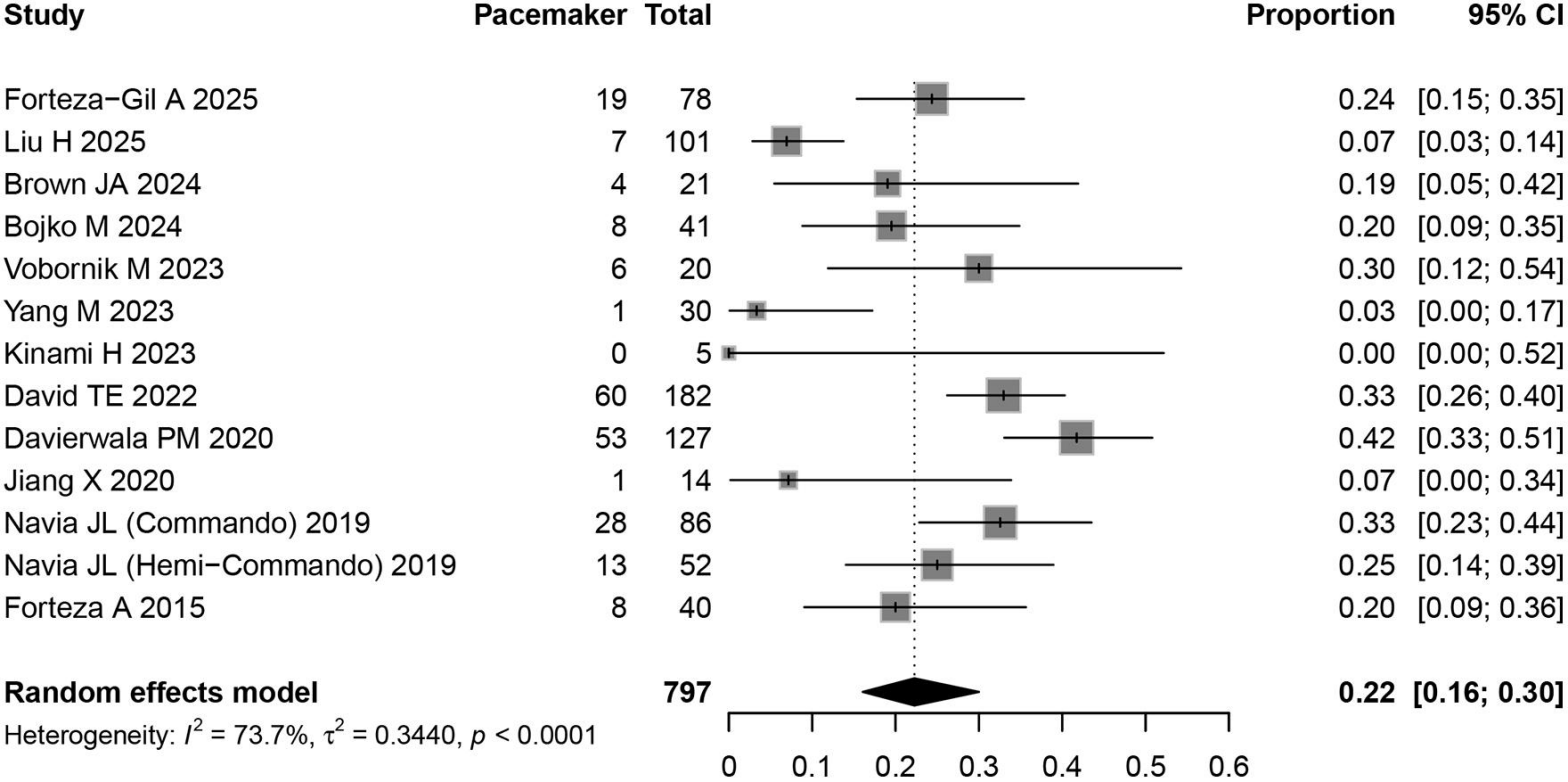
Forest plot of the pooled permanent pacemaker rate after Commando and Hemi-Commando procedures. Squares represent individual study proportions (size proportional to weight); horizontal lines indicate 95% confidence intervals; the diamond represents the random-effects pooled estimate (22.3%, 95% CI: 16.1%–30.0%). Heterogeneity: *I²* = 73.7%, *p* < 0.001.

The pooled permanent pacemaker rate under the random-effects model was 22.3% [95% confidence interval (CI): 16.1%–30.0%; Figure 2]. Heterogeneity was substantial (*I²* = 73.7%, ***τ*²** = 0.344, *p* < 0.001). The fixed-effect model yielded a higher estimate (28.8%, 95% CI: 25.6%–32.3%). Detailed meta-analysis results are presented in Table 2.

**Table 2:** Meta-analysis results for pacemaker implantation after commando procedure (random-effects model).

Analysis	No. of data points	Total patients	Pacemaker events	Pooled rate (95% CI)	*I²* (%)	*τ²*	*p* for heterogeneity
Overall	13	797	208	22.3 (16.1−30.0)	73.7	0.344	<0.001
By procedure type							
Commando	7	531	162	28.3 (21.2–36.7)	61.2	0.148	0.017
Hemi-Commando	1	52	13	25.0 (15.1–38.4)	—	—	—
Chimney Commando	2	131	8	6.3 (3.2–12.1)	0	0	0.48
Mixed (Commando + Hemi)	2	98	25	25.6 (17.9–35.1)	0	0	0.61
Pediatric	1	5	0	8.3 (0.5–62.2)*	—	—	—

* Value is imprecise due to zero events and small sample size.

### Subgroup analysis

**Figure 3:**
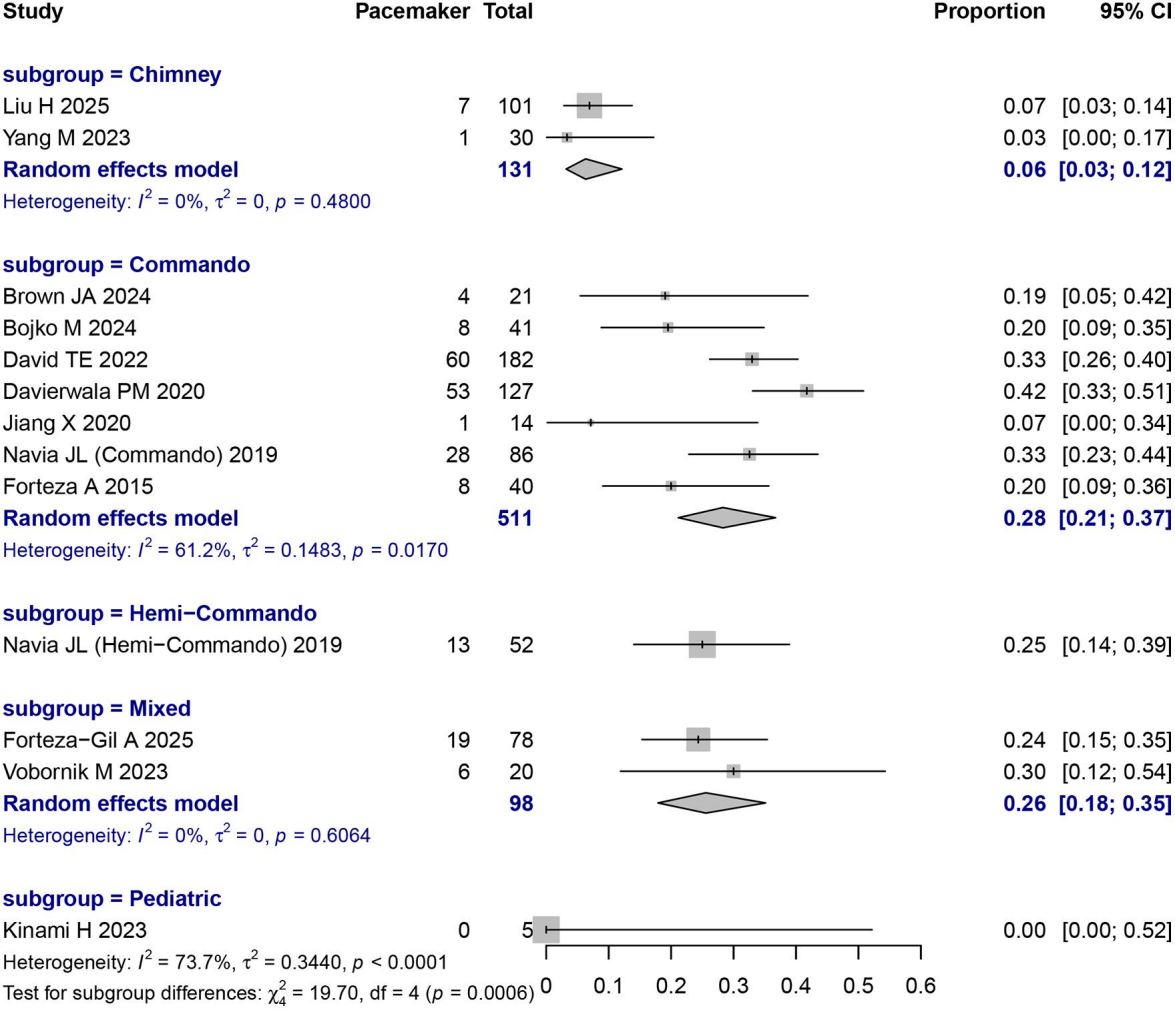
Subgroup forest plot stratified by procedure type. Random-effects models were used for each subgroup. The Chimney Commando subgroup showed a significantly lower pooled rate (6.3%, 95% CI: 3.2%–12.1%) than the Commando subgroup (28.3%, 95% CI: 21.2%–36.7%). Test for subgroup differences: *p* < 0.001.

Subgroup analysis by procedure type (Figure 3) showed that the Chimney Commando modification (2 data points, *n* = 131) had a significantly lower pooled rate of 6.3% (95% CI: 3.2%–12.1%) with no heterogeneity (*I²* = 0%). The traditional Commando procedure (7 data points, *n* = 511) had a pooled rate of 28.3% (95% CI: 21.2%–36.7%; *I²* = 61.2%). Mixed procedures (Commando + Hemi, 2 data points) gave 25.6% (95% CI: 17.9%–35.1%), and the single Hemi-Commando study gave 25.0% (95% CI: 15.1%–38.4%). The pediatric study (*n* = 5) had zero events. The test for subgroup differences was statistically significant (*p* < 0.001).

### Sensitivity analysis

Leave-one-out analysis showed that the pooled rate ranged from 20.7% (after excluding Davierwala 2020) to 26.5% (after excluding Liu H 2025), all within the original 95% CI (Supplementary Table S4). Excluding small studies (*n* < 20), the pediatric study, or extreme-rate studies did not materially change the estimate (range: 20.7%–23.7%). Heterogeneity decreased when Davierwala 2020 (*I²* = 66.4%) or Liu H 2025 (*I²* = 57.7%) was removed (detailed in Supplementary Table S4).

### Publication bias

**Figure 4:**
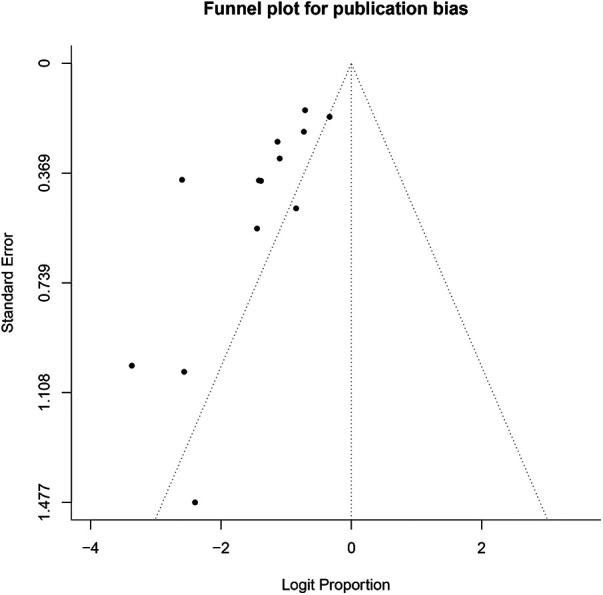
Funnel plot for publication bias. Each circle represents a study. Egger's test: *p* = 0.0041.

Egger's test suggested the presence of small-study effects (*t* = −3.61, *df* = 11, *p* = 0.0041). The funnel plot (Figure 4) showed some asymmetry, which may be explained by the substantial clinical and methodological heterogeneity.

## Discussion

This systematic review and meta-analysis provides the first meta-analysis specifically dedicated to permanent pacemaker implantation after Commando and Hemi-Commando procedures, providing an updated and focused estimate. The pooled rate of 22.3% (95% CI: 16.1%–30.0%) indicates that for every five patients undergoing this complex surgery, one will require a permanent pacemaker. This finding is a critical reference for preoperative risk-benefit analysis and informed consent discussions with patients. The risk is substantially higher than that reported for isolated aortic valve replacement (2%–5%) or isolated mitral valve surgery (3%–8%) (19, 20), underscoring the need for tailored preoperative counseling when planning Commando surgery. The clinical importance of avoiding permanent pacemaker implantation after cardiac surgery is underscored by a recent meta-analysis demonstrating that postoperative pacemaker dependence is associated with a 22% increase in long-term all-cause mortality (21). Our estimate is comparable to the 25.1% reported in a previous meta-analysis that included a broader range of outcomes (5), confirming the robustness of this finding.

The most clinically relevant finding is the striking difference between surgical modifications. The Chimney Commando technique, which uses a tongue-shaped vascular graft to enlarge both annuli while preserving the aorto-mitral geometry, was associated with a pacemaker rate of only 6.3% (95% CI: 3.2%–12.1%). This contrasts sharply with the traditional Commando procedure (28.3%) and the Hemi-Commando (25.0%). The lower rate may be attributed to reduced tension on the conduction system and improved anatomical alignment of the prosthetic valves. Contemporary large-center experience has also demonstrated the feasibility of the Commando procedure in complex scenarios such as anterior mitral annular calcification (22).

### Implications for clinical practice

These preliminary findings suggest that the Chimney modification might offer a lower pacemaker rate than the traditional Commando procedure, but this evidence comes from only two small case series (*n* = 131) and must be interpreted as hypothesis-generating. The Chimney technique cannot be routinely recommended at this time; it may be considered only in carefully selected patients (e.g., those with small annuli or undergoing redo surgery) and should be evaluated further in prospective comparative studies.

### Impact of active infection and tissue friability on conduction injury

In patients with infective endocarditis (IE)—the most common indication for the Commando procedure—active infection and associated tissue friability may independently increase the risk of atrioventricular conduction injury. The inflammatory response in IE leads to oedema, necrosis, and weakening of the intervalvular fibrous body and surrounding structures, making the conduction system more vulnerable to mechanical trauma during debridement and suture placement. Moreover, the need for extensive resection of infected tissue often removes the natural tissue barriers that protect the conduction fibres. These factors may contribute to the high pacemaker rate observed in endocarditis-related studies (up to 41.7% in Davierwala 2020) and should be considered when interpreting the pooled estimate. Surgeons operating in the setting of active IE should be particularly vigilant about preserving the atrioventricular conduction axis, and consider technical modifications (e.g., the Chimney technique) that minimize tension on the fibrous skeleton (3, 9).

Heterogeneity was substantial (*I²* = 73.7%), which is expected given the diversity of indications, surgical eras, sample sizes, and definitions of pacemaker implantation. Sensitivity analyses confirmed that no single study dominated the results, and the overall estimate remained stable across various exclusion criteria.

## Limitations

This meta-analysis has several limitations that should be considered when interpreting the results.

First, the retrospective, non-randomized nature of all included studies introduces selection bias and information bias. Patients undergoing Commando surgery at tertiary referral centres may differ systematically from those treated conservatively or at lower-volume centres, potentially inflating the observed pacemaker rate. Unmeasured confounders—including surgeon experience, surgical era, and institutional protocols—cannot be adjusted for in a single-arm meta-analysis. Consequently, our pooled estimate of 22.3% reflects observational evidence and should be applied cautiously in clinical decision-making.

Secondly, substantial heterogeneity persisted even after subgroup analysis (*I²* = 73.7% overall). This heterogeneity is likely driven by differences in surgical indications (e.g., endocarditis vs. calcific degeneration), surgical eras, sample sizes, and definitions of the time window for pacemaker ascertainment (in-hospital only vs. 30-day vs. entire follow-up). While we explored procedure type as a source of heterogeneity, other unmeasured factors (e.g., myocardial protection strategies) may also contribute. Future studies should standardize outcome definitions to reduce heterogeneity.

Third, publication bias cannot be ruled out (Egger's test *p* = 0.0041). Although funnel plot asymmetry may also reflect true heterogeneity, it is possible that smaller studies with higher pacemaker rates were preferentially published, potentially inflating our pooled estimate. Fourth, and most importantly, the comparison for the Chimney Commando modification derives from only two studies with a combined sample size of 131. Consequently, our finding of a lower pacemaker rate (6.3%) should be interpreted as hypothesis-generating rather than definitive. Future large-scale, preferably multicenter, prospective registries or comparative studies are urgently needed to validate these preliminary results and establish the long-term safety and efficacy of the Chimney technique. Additionally, most included studies did not report the specific indication for pacemaker implantation (e.g., complete AV block vs. sinus node dysfunction), which limits our ability to assess the clinical relevance of the implanted pacemakers and may introduce further heterogeneity.

Fifth, we did not assess the certainty of evidence using GRADE, as the included studies were all case series without comparators. Therefore, the pooled estimates represent the best available evidence but should be applied cautiously in clinical decision-making.

Sixth, we did not perform meta-regression to identify predictors of pacemaker implantation because the limited number of studies (only 7 in the Commando subgroup) would have resulted in underpowered and potentially spurious findings.

These findings are hypothesis-generating and require validation in larger, multicenter studies. Notably, recent meta-analyses published in this journal have addressed related topics in the field of valve surgery (23) and pacemaker risk prediction (24), highlighting the broader interest in this clinical problem.

## Conclusions

The Commando procedure carries a high risk of permanent pacemaker implantation (22.3%). Preliminary evidence suggests that the Chimney Commando modification is associated with a substantially lower risk (6.3% vs. 28.3%). For clinical practice, these preliminary findings suggest that the Chimney technique may be a reasonable option in carefully selected patients with small annuli or those undergoing redo surgery, where conduction injury risk is highest. However, given the limited number of studies (only two small case series), these results should be considered hypothesis-generating. Future prospective comparative studies and multicenter registries are urgently needed to confirm the long-term benefits of this technique. Meanwhile, surgeons should integrate these findings into preoperative patient counseling, particularly regarding the risk of permanent pacemaker dependence after Commando surgery.

## Data Availability

The data and code for this meta-analysis have been deposited in ScienceDB (DOI: 10.57760/sciencedb.37979). Further requests can be sent to Yong Cao, 118811825618@163.com.
