# Supplementary material for: Permanent pacemaker rate following Commando and Hemi-Commando procedures: a systematic review and meta-analysis
Source: Front Cardiovasc Med. 2026 Jun 23;13:1854238. doi: 10.3389/fcvm.2026.1854238 (PMC13337437; doi:10.3389/fcvm.2026.1854238)
Supplement: Supplementary file 2 [file Table1.docx]

### **Supplementary Table S1. Complete search strings for each database**

| Database | Search date | Search string |
| --- | --- | --- |
| PubMed | April 2, 2026 | ("commando procedure"[Title/Abstract] OR "commando operation"[Title/Abstract] OR "UFO procedure"[Title/Abstract] OR "intervalvular fibrous body reconstruction"[Title/Abstract] OR "aortic mitral curtain reconstruction"[Title/Abstract] OR "hemi-commando"[Title/Abstract] OR "modified commando"[Title/Abstract] OR "root-commando"[Title/Abstract]) |
| Embase | April 2, 2026 | commando:ti,ab,kw OR 'hemi-commando':ti,ab,kw OR 'modified commando':ti,ab,kw OR 'root-commando':ti,ab,kw OR 'ufo procedure':ti,ab,kw OR 'intervalvular fibrous body reconstruction':ti,ab,kw |
| Web of Science | April 2, 2026 | "commando procedure" OR "commando operation" OR "UFO procedure" OR "hemi-commando" OR "modified commando" OR "root-commando" OR "intervalvular fibrous body reconstruction" (Topic) |
| Cochrane CENTRAL | April 2, 2026 | "commando procedure" OR "commando operation" OR "hemi-commando" OR "modified commando" |
