# Supplementary material for: Permanent pacemaker rate following Commando and Hemi-Commando procedures: a systematic review and meta-analysis
Source: Front Cardiovasc Med. 2026 Jun 23;13:1854238. doi: 10.3389/fcvm.2026.1854238 (PMC13337437; doi:10.3389/fcvm.2026.1854238)
Supplement: Supplementary file 3 [file Table2.docx]

### **Supplementary Table S2. Baseline characteristics of included studies (pooled or range)**

| Characteristic | Value (range or pooled) | Number of studies reporting |
| --- | --- | --- |
| Mean age (years) | 52 – 67 (range across studies) | 12 / 12 |
| Male sex (%) | 31.7% – 90.0% (range) | 12 / 12 |
| Infective endocarditis as indication (%) | 13% – 100% (range; 61% overall in studies reporting) | 12 / 12 |
| Prior cardiac surgery (%) | 7.1% – 82% (range; 69% overall in studies reporting) | 12 / 12 |
| Prosthetic valve endocarditis (%) | 0% – 80% (range; only in IE subgroup) | 10 / 12 |
| EuroSCORE II or logistic EuroSCORE (mean) | 7.4% – 53.0% (range) | 5 / 12 |
| STS score | Not reported | 0 / 12 |

**Note**: Not all studies reported every variable. Ranges reflect the minimum and maximum reported values across studies. Percentages are calculated from studies that provided the specific variable. EuroSCORE was reported in five studies (Liu 2025, Davierwala 2020, Forteza‑Gil 2025, Bojko 2024, Brown 2024); STS score was not reported in any included study.
