# Supplementary material for: Permanent pacemaker rate following Commando and Hemi-Commando procedures: a systematic review and meta-analysis
Source: Front Cardiovasc Med. 2026 Jun 23;13:1854238. doi: 10.3389/fcvm.2026.1854238 (PMC13337437; doi:10.3389/fcvm.2026.1854238)
Supplement: Supplementary file 5 [file Table4.docx]

****Supplementary Table S4. Sensitivity analyses for pacemaker implantation rate****

| Analysis | No. of data points | Total patients | Pooled rate (95% CI) | I² (%) | τ² |
| --- | --- | --- | --- | --- | --- |
| Primary (random‑effects) | 13 | 797 | 22.3% (16.1%-30.0%) | 73.7 | 0.344 |
| Fixed‑effect model | 13 | 797 | 28.8% (25.6%-32.3%) | — | — |
| Leave‑one‑out (range) | — | — | 20.7%-26.5% | — | — |
| Excluding small studies (n < 20) | 11 | 778 | 23.4% (17.0%-31.4%) | 76.2 | 0.326 |
| Excluding pediatric study | 12 | 792 | 22.6% (16.3%-30.5%) | 75.3 | 0.343 |
| Excluding Davierwala 2020 (highest rate) | 12 | 670 | 20.7% (15.0%-27.9%) | 66.4 | 0.269 |
| Excluding Yang 2023 (lowest rate) | 12 | 767 | 23.7% (17.5%-31.3%) | 72.3 | 0.288 |
